# Reliability and sensitivity of two whole-brain segmentation approaches included in FreeSurfer – ASEG and SAMSEG

**DOI:** 10.1016/j.neuroimage.2021.118113

**Published:** 2021-05-01

**Authors:** Donatas Sederevičius, Didac Vidal-Piñeiro, Øystein Sørensen, Koen van Leemput, Juan Eugenio Iglesias, Adrian V. Dalca, Douglas N. Greve, Bruce Fischl, Atle Bjørnerud, Kristine B. Walhovd, Anders M. Fjell, Alzheimers Disease Neuroimaging Initiative

**Affiliations:** a Center for Lifespan Changes in Brain and Cognition, University of Oslo, Pb. 1094, Blindern, Oslo 0317, Norway; b Division of Radiology and Nuclear Medicine, Oslo University Hospital, Norway; c Athinoula A. Martinos Center for Biomedical Imaging, Massachusetts General Hospital and Harvard Medical School, United States; d Department of Health Technology, Technical University of Denmark, Denmark; e Centre for Medical Image Computing, Department of Medical Physics and Biomedical Engineering, University College London, United Kingdom; f Computer Science and Artificial Intelligence Laboratory, MIT, United States

## Abstract

Accurate and reliable whole-brain segmentation is critical to longitudinal neuroimaging studies. We undertake a comparative analysis of two subcortical segmentation methods, Automatic Segmentation (ASEG) and Sequence Adaptive Multimodal Segmentation (SAMSEG), recently provided in the open-source neuroimaging package FreeSurfer 7.1, with regard to reliability, bias, sensitivity to detect longitudinal change, and diagnostic sensitivity to Alzheimer’s disease. First, we assess intra- and inter-scanner reliability for eight bilateral subcortical structures: amygdala, caudate, hippocampus, lateral ventricles, nucleus accumbens, pallidum, putamen and thalamus. For intra-scanner analysis we use a large sample of participants (*n* = 1629) distributed across the lifespan (age range = 4–93 years) and acquired on a 1.5T Siemens Avanto (*n* = 774) and a 3T Siemens Skyra (*n* = 855) scanners. For inter-scanner analysis we use a sample of 24 participants scanned on the day with three models of Siemens scanners: 1.5T Avanto, 3T Skyra and 3T Prisma. Second, we test how each method detects volumetric age change using longitudinal follow up scans (*n* = 491 for Avanto and *n* = 245 for Skyra; interscan interval = 1–10 years). Finally, we test sensitivity to clinically relevant change. We compare annual rate of hippocampal atrophy in cognitively normal older adults (*n* = 20), patients with mild cognitive impairment (*n* = 20) and Alzheimer’s disease (*n* = 20). We find that both ASEG and SAMSEG are reliable and lead to the detection of within-person longitudinal change, although with notable differences between age-trajectories for most structures, including hippocampus and amygdala. In summary, SAMSEG yields significantly lower differences between repeated measures for intra- and inter-scanner analysis without compromising sensitivity to changes and demonstrating ability to detect clinically relevant longitudinal changes.

## 1. Introduction

Automated techniques for whole-brain segmentation have become extremely useful in the study of a range of brain diseases and conditions, such as Alzheimer’s disease (AD) (Chételat, 2018), and also normal changes such as in development (Ostby et al., 2009) and aging (Wonderlick et al., 2009). Automated techniques enable processing of large numbers of magnetic resonance imaging (MRI) scans with limited operator investments, enabling detailed segmentations of brains from large-scale brain imaging initiatives. One of the most extensively used whole-brain segmentation approaches is Automatic Segmentation (ASEG) (Fischl et al., 2002), distributed as part of FreeSurfer (http://freesurfer.net/) (Fischl, 2012). FreeSurfer ASEG is a core tool in large-scale neuroimaging projects such as the UK Biobank (≈ 40.000 scans to date) (Alfaro-Almagro et al., 2018), ABCD (≈ 10.000 scans to date) (Hagler et al., 2019), ADNI (> 20.000 scans) (Jack et al., 2008), ENIGMA (> 50.000 scans) (Thompson et al., 2020), and Lifebrain (≈ 10.000 scans) (Walhovd et al., 2018). Although the accuracy of automated segmentation techniques such as ASEG is generally high and enables detection of longitudinal changes (Mulder et al., 2014; Worker et al., 2018), reports have suggested that segmentation accuracy may vary as a function of variables such as age (Wenger et al., 2014) and brain size (Herten et al., 2019; Schoemaker et al., 2016). Hence, continued efforts are undertaken to improve accuracy and reduce bias in the segmentations.

Similar to many other current whole-brain segmentation techniques, ASEG is based on supervised models of T1-weighted images. As signal intensities alone are not sufficient to distinguish between different neuroanatomical structures from a T1-weighted MRI, an atlas containing probabilistic information about the location of structures is used to determine the relationship between intensities and neuroanatomical labels in particular regions of the brain. The probabilistic atlas is generated from a set of manually labeled training images. The segmentation problem is then solved in a Bayesian framework in which local shape, position and appearance all contribute to the probability of a given label. Recently, an alternative approach was suggested - Sequence Adaptive Multimodal Segmentation (SAMSEG) – which uses generative parametric models (Puonti et al., 2013, 2016). Unlike ASEG, SAMSEG uses a mesh-based computational atlas combined with a Gaussian appearance model to achieve independence of specific image contrast by grouping together voxels with similar intensities (Van Leemput, 2009). SAMSEG is less computationally demanding than other iterative segmentation methods since no preprocessing is needed and only a single, efficient non-linear registration of the atlas to the target image is required. Moreover, bias field estimation and correction are done simultaneous with segmentation and non-linear registration. Nevertheless, SAMSEG resulted in accuracy comparable to ASEG and three other state-of-the-art methods in segmenting T1-weighted MRIs (Puonti et al., 2016). Since SAMSEG does not rely on the specific intensity profiles of a separate training data set, it yields consistent segmentations across scanner platforms and pulse sequences. SAMSEG is included as part of the recent FreeSurfer 7.1 release (released May 11th, 2020), which enables its general use in the neuroimaging community. Therefore, a thorough analysis is necessary to direct the choice between these two utilities provided in the same widely used package of FreeSurfer.

In the present study we undertake a thorough comparative analysis of SAMSEG and ASEG in terms of reliability, bias, sensitivity to longitudinal change, and clinical sensitivity. Longitudinal SAMSEG is used in the present study, which was not available at the time of the Puonti et al. (2016) study. First, we assess intra- and inter- scanner reliability. Second, since higher reliability could come at the cost of lower sensitivity to biologically meaningful change, we test how ASEG and SAMSEG are able to detect neuroanatomic volumetric change in longitudinal follow up scans. Finally, we test how sensitive each method is to clinically relevant change by comparing the annual rate of hippocampal atrophy in a group of cognitively normal older adults (CN), patients with mild cognitive impairment (MCI) and patients with AD.

## 2. Materials and methods

### 2.1. Datasets

#### 2.1.1. Lifespan scan-rescan dataset

**Fig. 1.**
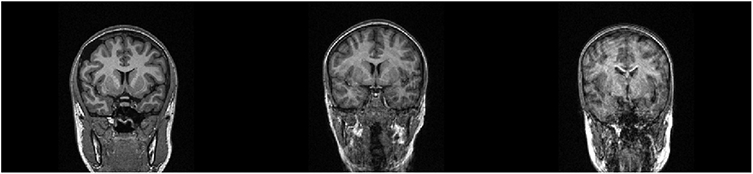
Examples of visual exclusion criterion. Left panel shows motion-free normal looking brains; center and right panels show images that have visible motion artefacts.

**Fig. 2.**
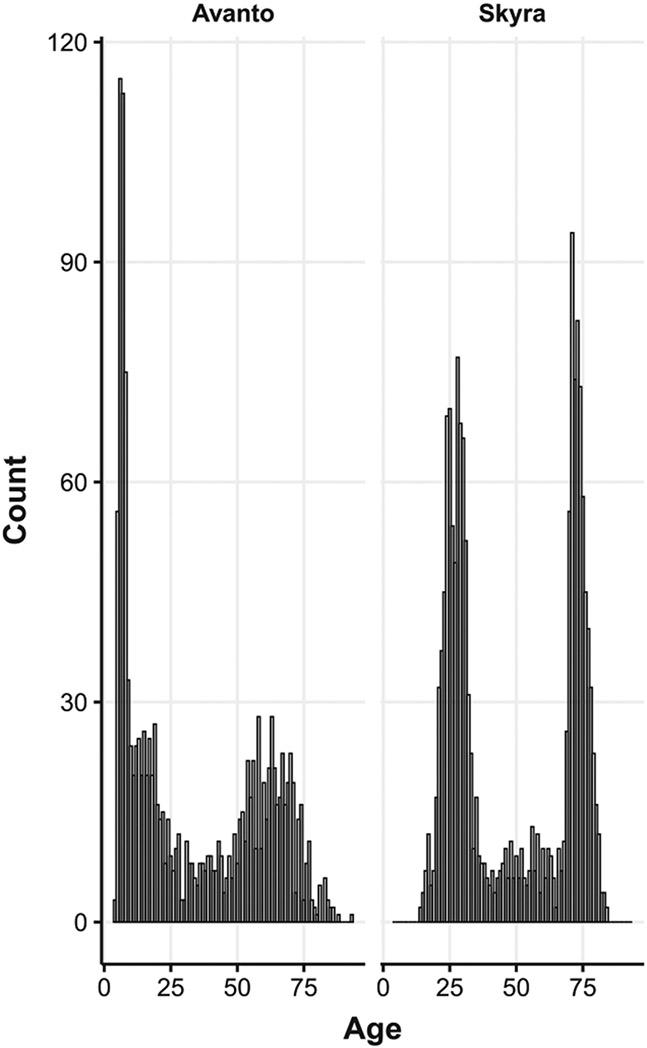
Age distributions of Avanto and Skyra datasets.

We use scan-rescan dataset selected from several ongoing projects at the Center for Lifespan Changes in Brain and Cognition (LCBC), University of Oslo, approved by the Regional Committees for Medical and Health Research Ethics South of Norway. Participants were cognitively healthy, and all participants or their guardian provided informed consent (for details, see e.g. (Walhovd et al., 2016)). Images were acquired using two models of Siemens MRI scanners (Siemens Medical Solutions, Erlangen, Germany) - 1.5T Avanto and 3T Skyra, at Rikshospitalet, Oslo University Hospital. A total of 890 participants (1643 sessions) and 887 participants (1739 sessions) were included in the initial within-session scan-rescan datasets for Avanto and Skyra scanners respectively. All images were visually inspected for motion artefacts, and sessions that had two images of no visual appearance of motion were included in further analysis. Fig. 1 illustrates examples of exclusion criterion. After discarding images with insufficient quality, the samples were reduced to 774 participants (427 females; 1362 sessions; age range = 4–93 years) for Avanto and 855 participants (563 females; 1646 sessions; age range = 14–84 years) for Skyra. Fig 2 summarizes age distributions of each scanner dataset. All data was acquired using Magnetization Prepared Rapid Gradient Echo (MPRAGE) sequence with parameters summarized in Table 1. The parameters for the scan-rescan datasets differed between the scanners but were identical for each session on the same scanner, except for the Skyra dataset where one image was acquired using parallel acquisition factor GRAPPA=1 and rescanned with GRAPPA=2. To acquire data with optimal comparability within each scanner, participants remained in the same position between scan and rescan acquisitions.

#### 2.1.2. Inter-scanner dataset

For inter-scanner dataset, we use a sample of 24 participants (19 females, age range between 20 and 36 years) scanned with three models of Siemens MRI scanners (Siemens Medical Solutions, Erlangen, Germany) on the same day - 1.5T Avanto, 3T Skyra and 3T Prisma, at Rikshospitalet, Oslo University Hospital. Table 1 summarizes MRI T1w pulse sequence parameters of each scanner.

#### 2.1.3. Lifespan longitudinal datasets

For longitudinal LCBC datasets, we select participants from the scan-rescan dataset who also have a follow-up visit: 491 participants of the Avanto scanner and 245 participants of the Skyra scanner. Each participant has two visits with the follow-up ranging from 1 to 10 years for the Avanto dataset and 1 to 5 years for the Skyra dataset.

#### 2.1.4. Clinical sensitivity dataset

In addition to the longitudinal LCBC datasets, we also include scans from the Alzheimer’s disease Neuroimaging Initiative (ADNI) database (adni.loni.usc.edu). The ADNI was launched in 2003 as a public-private partnership, led by Principal Investigator Michael W. Weiner, MD. The primary goal of ADNI has been to test whether serial MRI, positron emission tomography, other biological makers, and clinical and neuropsychological assessment can be combined to measure the progression of MCI and early AD. For up-to-date information, see www.adni-info.org. For our study, we randomly select three groups of participants with similar age distributions: CN, MCI and AD. Each group consist of 20 participants. The selected sample of ADNI data has been acquired at different sites using a Siemens Avanto 1.5T MRI scanner and MPRAGE sequence: TR = 2400 ms, TE = 3.54 ms, TI = 1000 ms, flip angle = 8°, voxel size = 1.25 × 1.25 × 1.2 mm^3^, 192 × 192 acquisition matrix, 160 slices, 180 Hz pixel bandwidth, GRAPPA = 1, 8 channel matrix coil. Each participant has two visits with a follow-up ranging from 6 months to 2 years for each group.

### 2.3. MRI processing

Due to the non-linearity of the magnetic fields from the imaging gradient coils, we first preprocess images to reduce geometrical variability of the same participants’ brains between sessions. This is achieved by obtaining scanner-specific spherical harmonics expansions that represent the gradient coils (Jovicich et al., 2006).

We use two fully automated subcortical segmentation methods FreeSurfer v7.1 ASEG and SAMSEG to process MRI data and measure volumes of eight bilateral brain structures of interest: amygdala, caudate, hippocampus, lateral ventricles, nucleus accumbens, pallidum, putamen and thalamus. Briefly, the FreeSurfer ASEG pipeline includes Talairach transformation, intensity correction, the removal of nonbrain tissues and volumetric brain segmentation based upon the existence of an atlas containing information on the location of structures, whereas SAMSEG utilizes a *mesh-based* atlas and a Bayesian modeling framework to obtain volumetric segmentations without the need for skull-stripping. Moreover, SAMSEG does the bias field estimation and correction simultaneous with segmentation and non-linear registration which is not the case for ASEG where each step is performed separately. Both methods are fully automated and model-based that use a pre-built probabilistic atlas prior from 39 to 20 subjects, respectively. The 20 subjects used for SAMSEG are a subset of the 39 used for ASEG.

To extract reliable volume estimates, we process all datasets with the longitudinal stream in FreeSurfer ASEG and SAMSEG. For FreeSurfer ASEG, an unbiased within-subject template space and image (Reuter and Fischl, 2011) is created using robust, inverse consistent registration (Reuter et al., 2010). Several processing steps, such as skull stripping, Talairach transforms, atlas registration, and spherical surface maps and parcellations are then initialized with common information from the within-subject template, significantly increasing reliability and statistical power (Reuter et al., 2012). Longitudinal SAMSEG is based on a generative model of longitudinal data (Iglesias et al., 2016). In the forward model, a subject-specific atlas is obtained by generating a random warp from the usual population atlas, and subsequently each time point is again randomly warped from this subject-specific atlas. Bayesian inference is used to obtain the most likely segmentations, with the intermediate subject-specific atlas playing the role of latent variable in the model, whose function is to ensure that various time points have atlas warps that are similar between themselves, without having to define a priori what these warps should be similar to.

### 2.4. Statistical analysis

#### 2.4.1. Scan-rescan reliability

We use multiple statistical approaches to describe and evaluate the magnitude of intra- and inter-scanner variability between repeated measurements. We calculate the absolute symmetrized percent difference (ASPD) as follows:

$$ASPD(L_{1},L_{2})=\frac{2|V(L_{1})-V(L_{2})|}{V(L_{1})+V(L_{2})}\times 100\%,$$

where *L*_1_ and *L*_2_ are the segmented labels of the same structure but of different images and *V*(*L*) is the volume of the label. ASPD value of 0 indicates a perfect replicability, with increasing values indicating less reliable repeated measurements. We use Generalized additive models (GAM) (Wood, 2017) to characterize volume estimation variability trends of subcortical structures across the lifespan. GAMs are generalized linear models in which the predictors depend linearly or non-linearly on some smooth non-linear functions (Hastie and Tibshirani, 1990). The smooth functions are estimated from the data and enable a flexible smooth curve fitting across the lifespan. In addition to ASPD, we also calculate Dice scores (Dice, 1945), intraclass correlation coefficients (ICC) (McGraw and Wong, 1996; Koo and Li, 2016) and Bland-Altman plots (Bland and Altman, 1986). For ICC we use a 2-way mixed-effects model, single measurement and absolute agreement ICC form.

#### 2.4.2. Sensitivity to longitudinal change

First, to assess whether the estimated lifespan trajectories of the subcortical volumes differ depending on segmentation method, we use General Additive Mixed Models (GAMM) (Wood, 2017). In contrast to GAMs which treat each observation as independent, GAMMs take longitudinal information into account by explicitly modeling the correlation between repeated measurements of the same subject, yielding a model which captures cross-sectional and longitudinal information. Second, to assess longitudinal changes, we use the annualized percentage change (APC) values between the baseline and the follow-up visits for all participants with two scans separated by one or more years. We compare APC values for each segmentation method with paired samples t-tests. We divide the sample into development (< 20 years), adulthood (between 20 and 60 years) and aging (> 60 years) and compare APCs across age groups using t-tests and Cohen’s D. Cohen’s D is an effect size used to indicate the standardized difference between two means. Third, to address the clinical sensitivity of each segmentation method, we compute APC for the hippocampus for ADNI subjects, and assess differences in APC between groups (NC vs. MCI vs. AD) using Cohen’s D. Finally, we use Receiver Operating Characteristic (ROC) curves and Area Under the Curve (AUC) to address the classification sensitivity based on the APC values of the longitudinal hippocampus estimates in different groups.

All statistical analyses described above is done using R statistical software package v3.6.3 (R Core Team, 2020) and its related packages: *mgcv* (Wood, 2017), *ggplot2* (Wickham, 2016), *ggpubr* (Kassambara, 2020), *cowplot* (Wilke, 2019), *irr* (Gamer et al., 2019), *effsize* (Torchiano, 2020) and *dplyr* (Wickham et al., 2020).

## 3. Results

### 3.1. Scan-rescan reliability

**Fig. 3.**
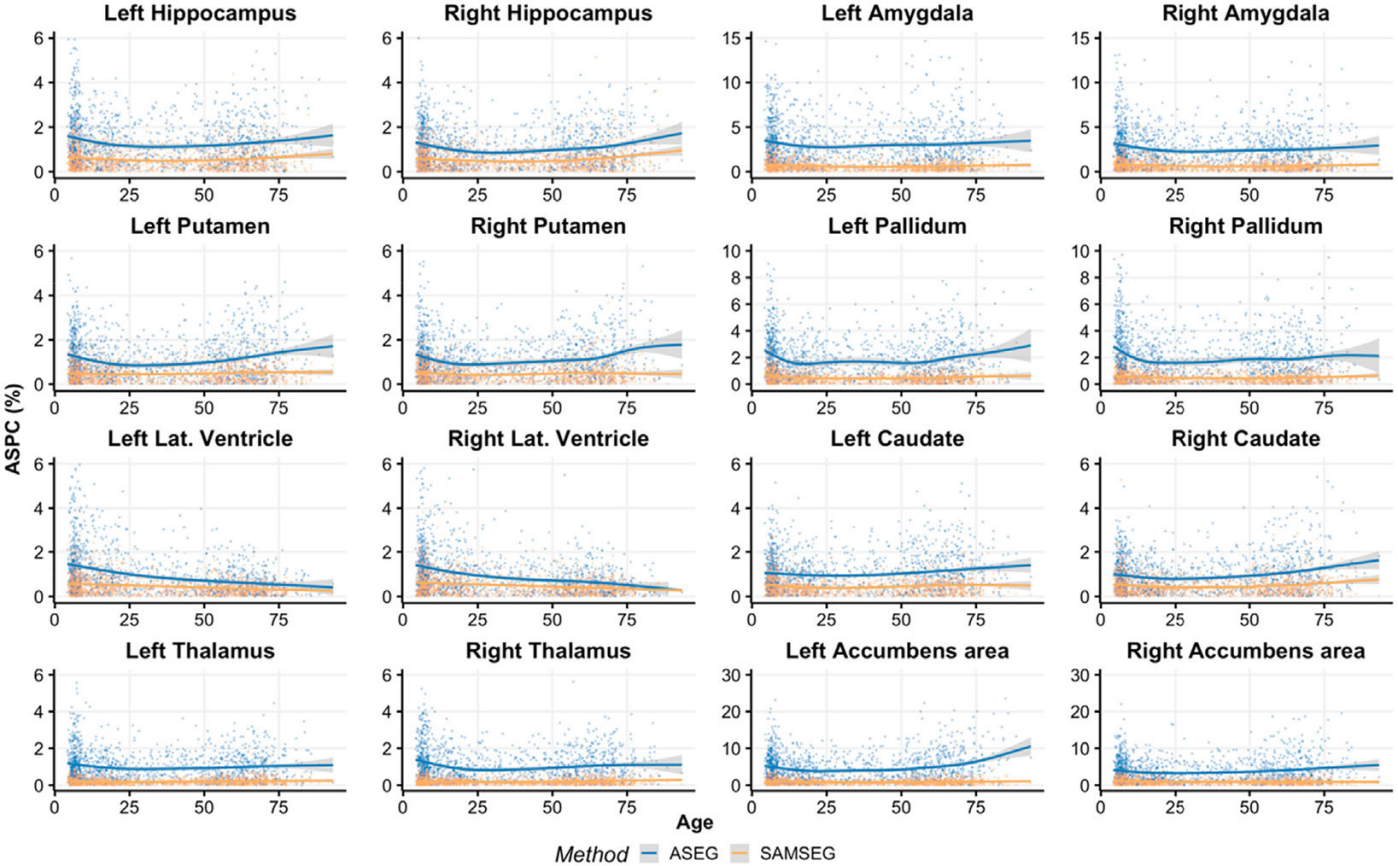
ASPC values across the lifespan for the Avanto dataset. Age-related trends are shown by the GAM curves.

**Fig. 4.**
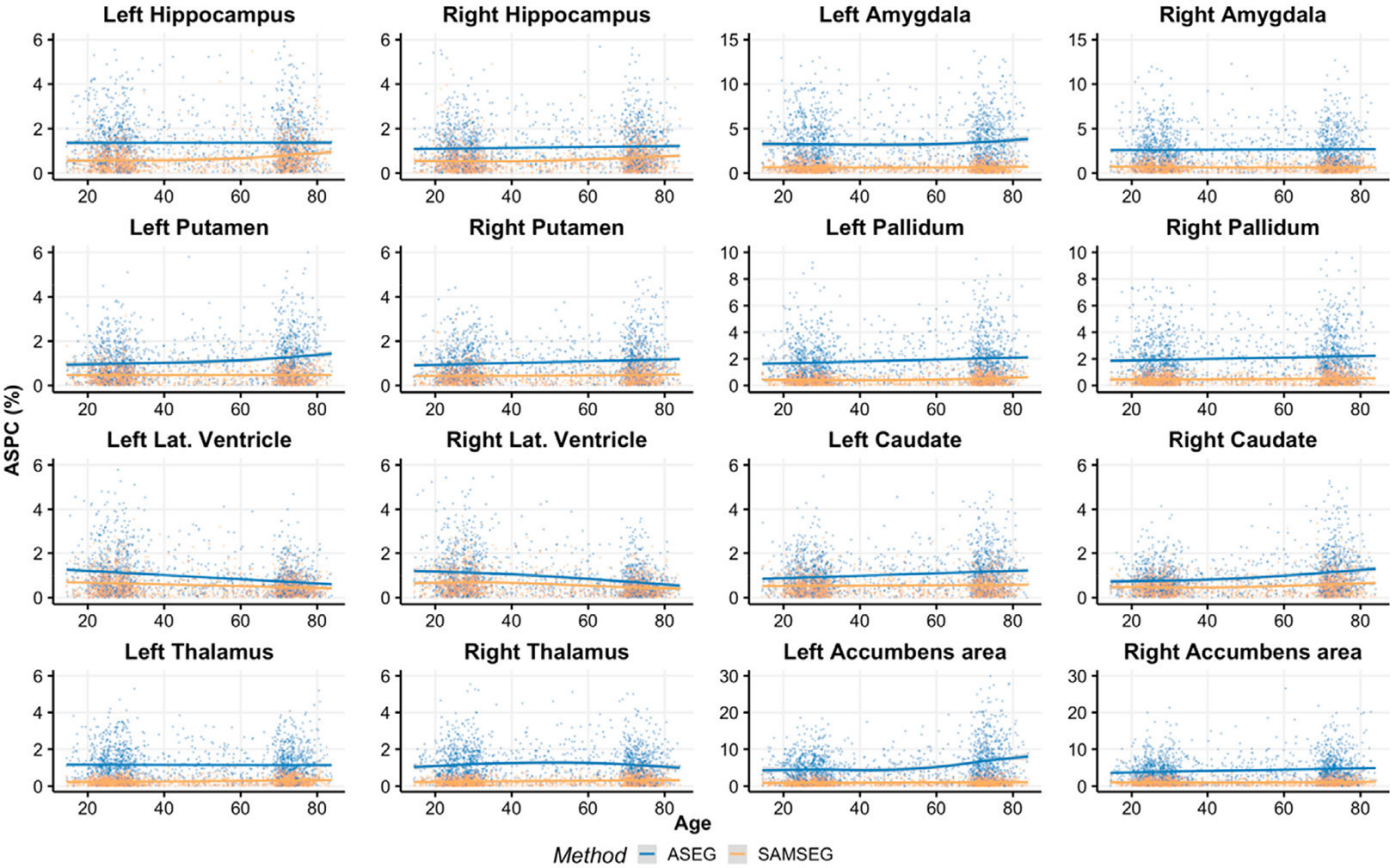
ASPC values across the lifespan for the Skyra dataset. Age-related trends are shown by the GAM curves.

Fig. 3 and Fig. 4 show volume estimation differences between repeated intra-scanner acquisitions across the lifespan for the Avanto and Skyra datasets respectively. Although most of the subcortical structures indicate relatively flat lifespan trends, small deviations are observed in the Avanto dataset for the young children group (age < 10 years) when using ASEG. This is not present in the Skyra dataset as it does not include this age group. SAMSEG volumetric estimates are significantly lower (paired samples *t*-test, *p* < 0.05) for both datasets and all structures across the lifespan, see appendix (Table A.1) for summary statistics which also indicate lower standard deviations for SAMSEG.

**Fig. 5.**
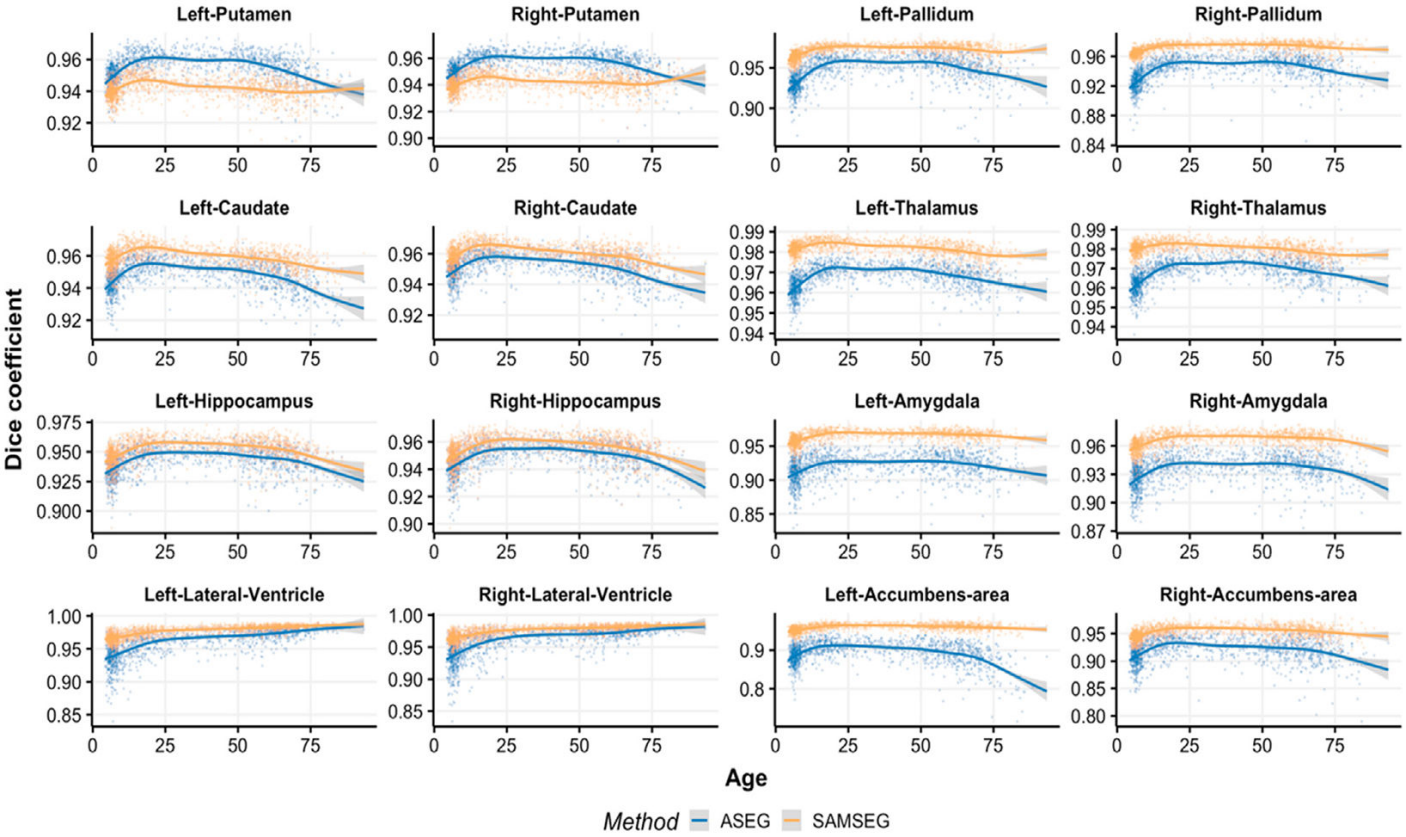
Dice coefficients across the lifespan for the Avanto dataset. Age-related trajectories are shown by the GAM curves. The y-axis scale varies across plots to enable easier evaluation of age-trends.

**Fig. 6.**
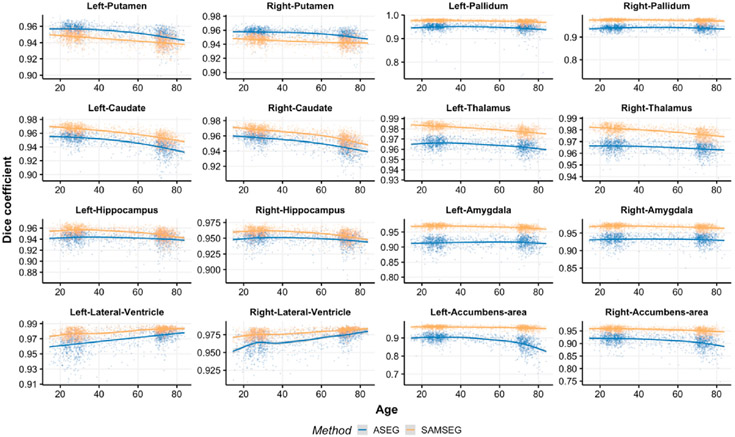
Dice coefficients across the lifespan for the Skyra dataset. Age-related trajectories are shown by the GAM curves. The y-axis scale varies across plots to facilitate easier evaluation of age-trends.

Fig. 5 and Fig. 6 indicate spatial overlap similarity in terms of dice scores for the Avanto and Skyra datasets respectively. Most of the structures show inverted u-shape trajectories except the lateral ventricles which demonstrate almost linearly increasing reliability with aging. ASEG yields significantly higher spatial agreement for putamen whereas the rest of the spatial overlaps are significantly better for SAMSEG (paired samples *t*-test, *p* < 0.01). The largest improvements are demonstrated for amygdala, pallidum and nucleus accumbens. In general, all Dice scores are high for both the segmentation methods indicating a good spatial agreement between segmented volumes.

**Fig. A.1.**
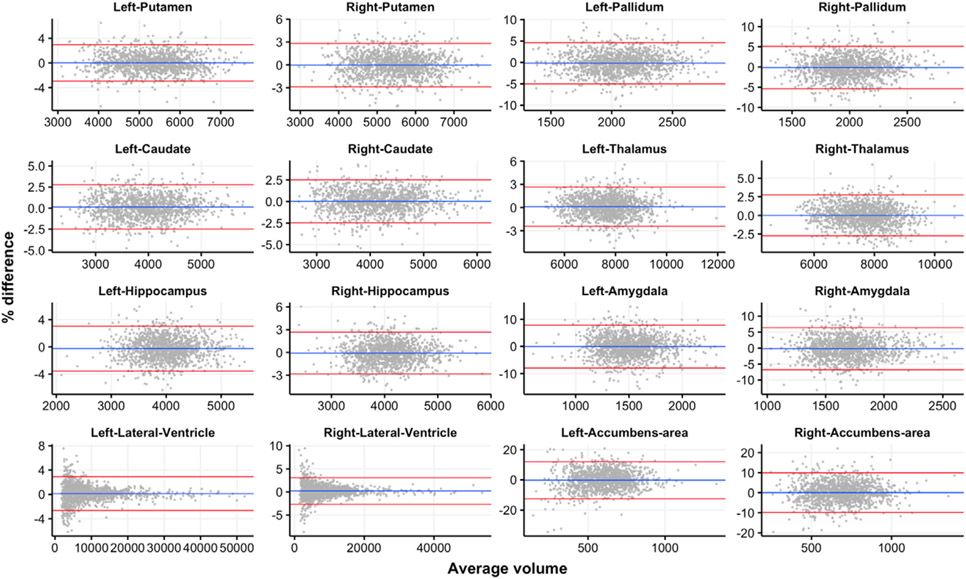
Bland-Altman plots for the Avanto dataset and ASEG segmentation method. Limits of agreement (average difference ± 1.96 standard deviation of the difference) are shown by the red lines.

**Fig. A.2.**
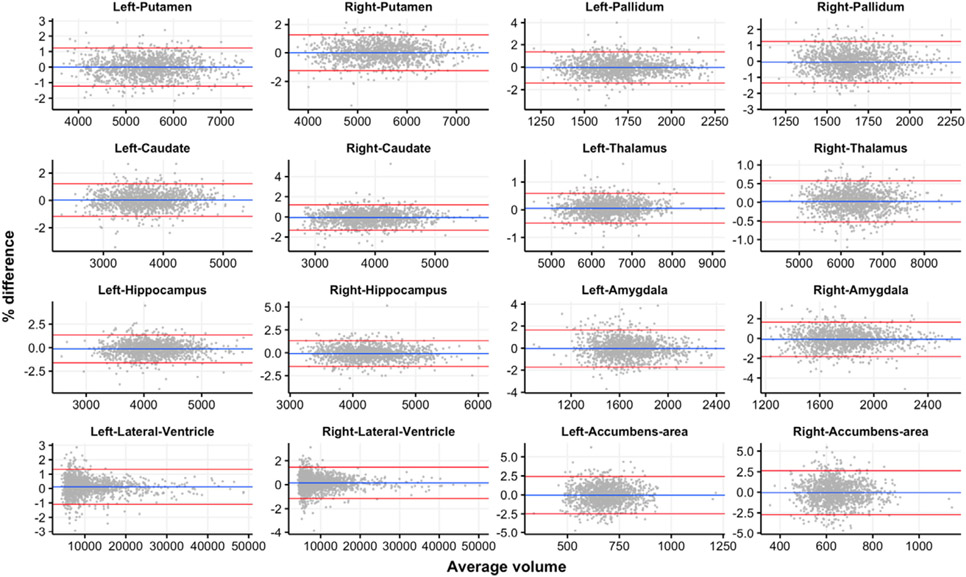
Bland-Altman plots for the Avanto dataset and SAMSEG segmentation method. Limits of agreement (average difference ± 1.96 standard deviation of the difference) are shown by the red lines.

We also compute ICC to assess the agreement between the repeated measurements for each scanner dataset and segmentation method. Although we find the reliability of the repeated measurements very high (ICC > 0.95) for both methods, SAMSEG results in significantly higher (*p* < 0.01) ICC values for all subcortical structures. Bland-Altman plots of both methods do not indicate bias towards the estimated structure size, see appendix (Fig. A.1 and Fig. A.2). However, despite consistent volumetric estimations regardless of the structure size, the limits of agreement *(average difference* ± *1.96 standard deviation of the difference)* are in favor of SAMSEG.

### 3.2. Inter-scanner differences

**Fig. 7.**
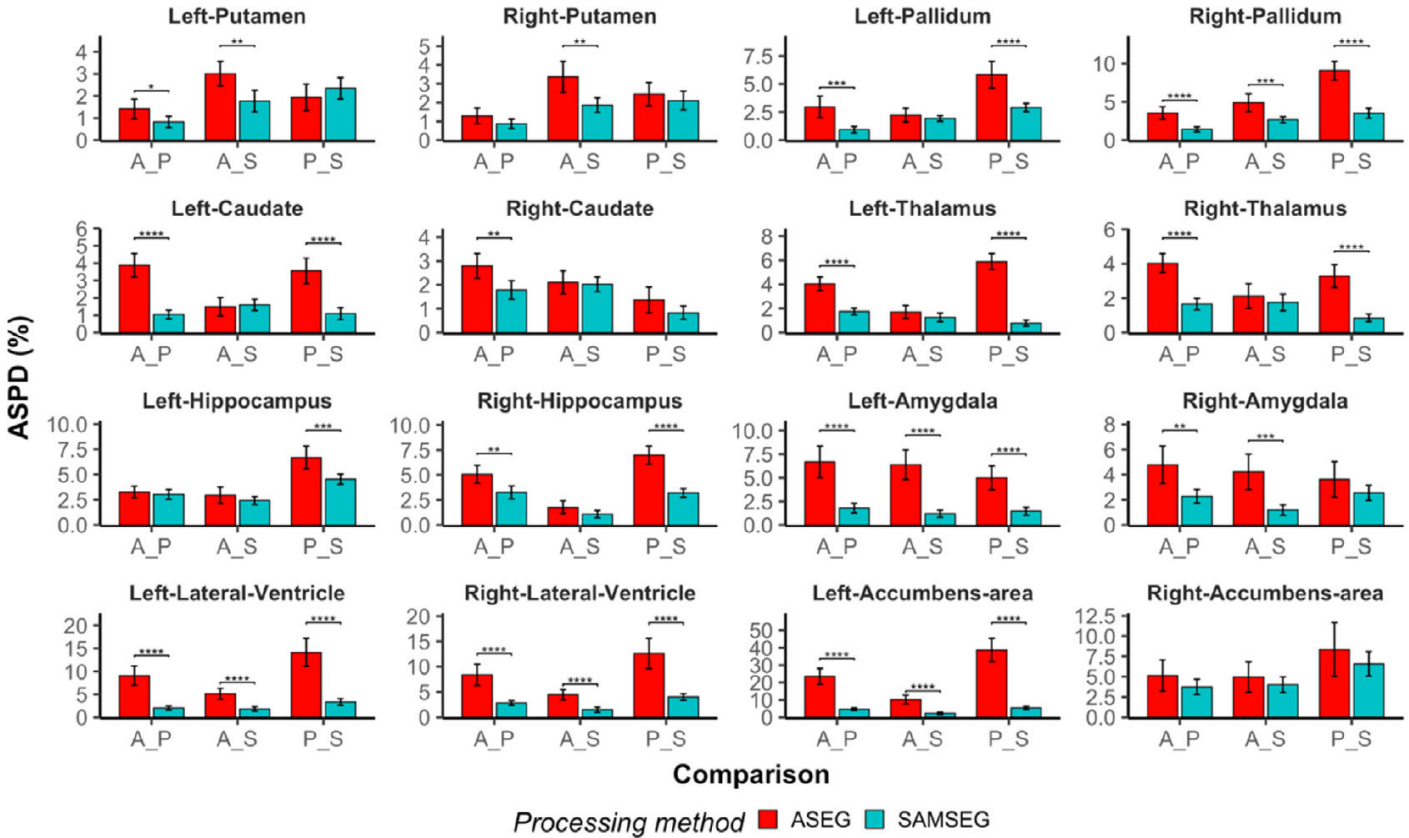
Bar plots of ASPD values for inter-scanner comparisons. X-axis abbreviations: Avanto vs. Prisma (A vs. P), Avanto vs. Skyra (A vs. S) and Prisma vs. Skyra (P vs. S). Significant differences between segmentation methods are indicated by horizontal lines with significance codes of the p-values above: 0.0001 ‘ * * * * ’, 0.001 ‘ * * * ’, 0.01 ‘ * * ’, 0.05 ‘ * ’.

**Fig. 8.**
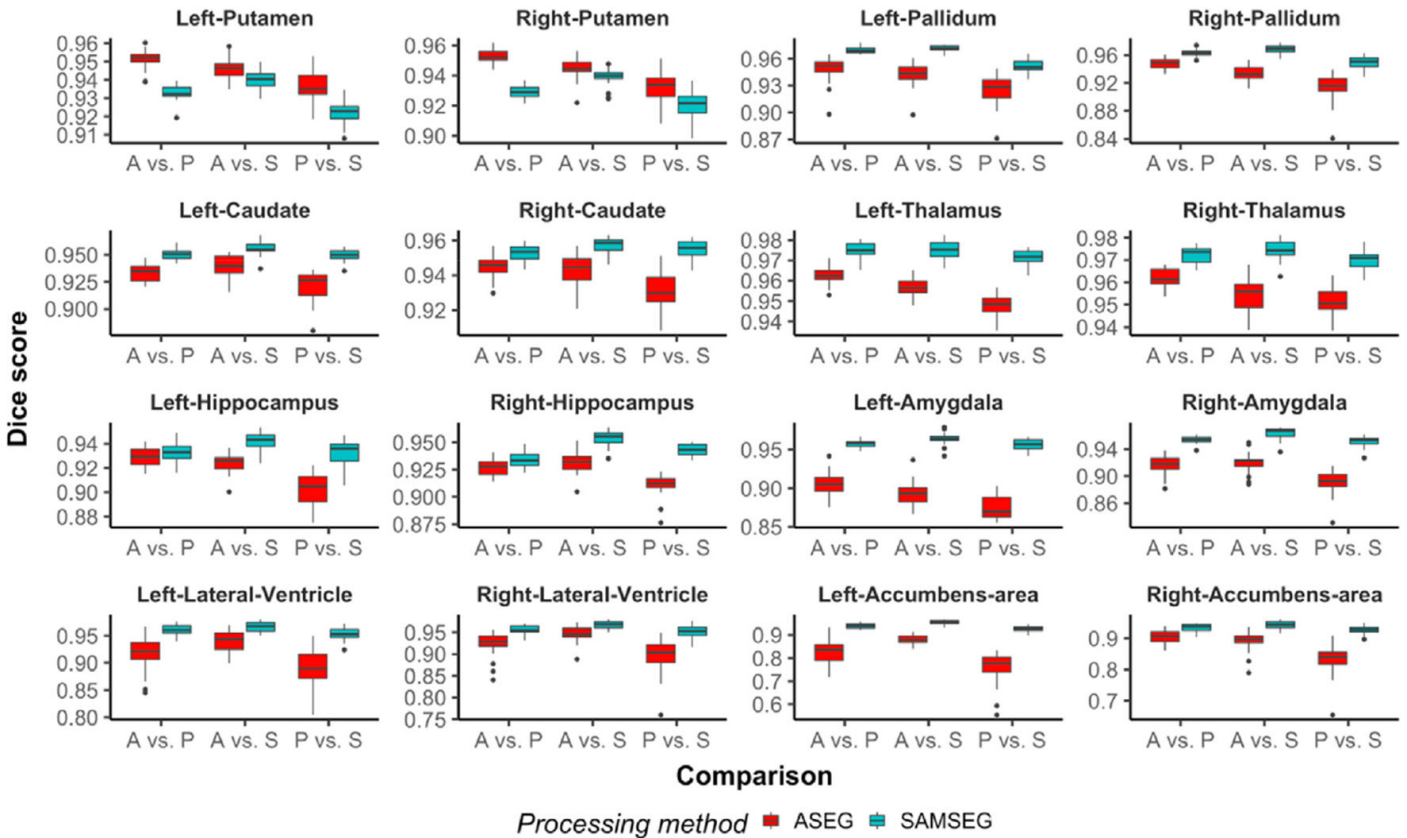
Box plots of Dice scores for inter-scanner comparisons. X-axis abbreviations: Avanto vs. Prisma (A vs. P), Avanto vs. Skyra (A vs. S) and Prisma vs. Skyra (P vs. S). All differences between segmentation methods are significant (*p* < 0.05).

In Fig. 7, we present inter-scanner differences for three comparisons: Avanto vs. Prisma, Avanto vs. Skyra, and Prisma vs. Skyra. It is evident that the performance of both segmentation methods depends on the particular choice of comparison. Nevertheless, most of the estimated differences are in favor of SAMSEG, especially for amygdala, lateral ventricles and pallidum. A table of numerical results (means and standard deviations) is provided in the appendix (see Table A.2). Similar to scan-rescan reliability, spatial overlaps are also significantly better for SAMSEG except putamen, which has significantly better scores for ASEG, see Fig. 8.

### 3.3. Longitudinal changes

**Fig. 9.**
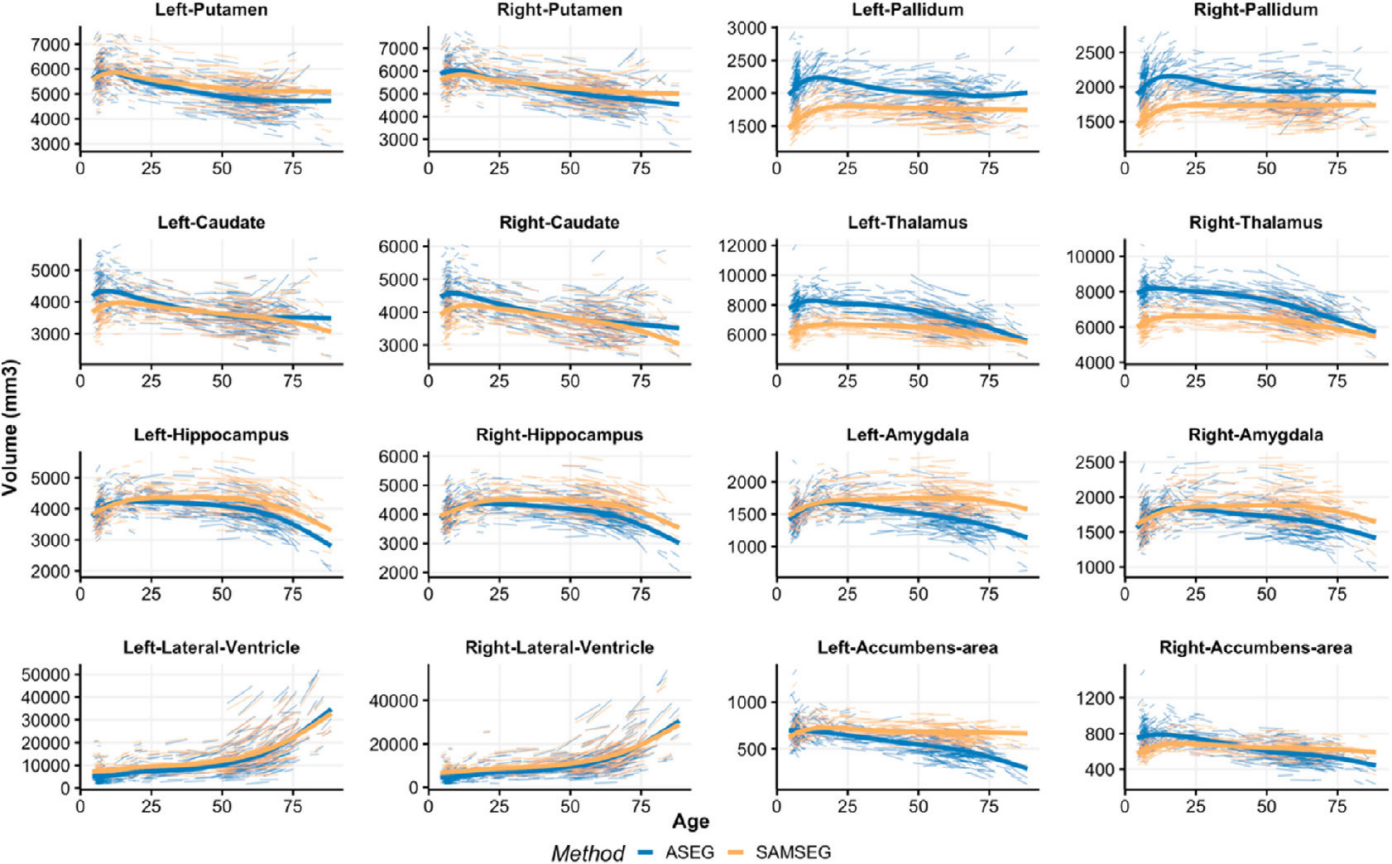
Lifespan trajectories for the Avanto dataset. The trajectories are estimated by GAMM and represent a combination of cross-sectional and longitudinal information.

SAMSEG’s higher intra- and inter-scanner reliability could be a result of a lower sensitivity to detect relevant changes in brain volumes. We, therefore, test the sensitivity of ASEG and SAMSEG to detect changes over time using longitudinal scans and previously documented effects. First, we run GAMMs to test whether ASEG and SAMSEG yields distinct estimated lifespan trajectories for the volume of each structure when both cross-sectional and longitudinal information is taken into account. For this, we use a part of the LCBC scan-rescan dataset where two observations separated by at least one year are available for each participant. Each volume’s trajectory is modelled as a function of age, which varies within each participant with more than one test occasion. The resulting curves thus take into account both observed within-participant change and between participant differences in age. Fig. 9 shows the estimated lifespan trajectories for each method for the longitudinal Avanto dataset. Although there are similarities in estimated age-trajectories between segmentation methods, there are also marked differences. Specifically, ASEG estimates more prominent age-effects for the hippocampus, amygdala and thalamus structures, with apparent volumetric reductions starting at a much earlier age compared to the SAMSEG results. We observe similar results for the Skyra dataset as well.

Next, we analyze change as indexed by the APC between time-points. We divide the sample into 3 age groups: development, adulthood and aging as described in Section 2.4.2. Table 2 summarizes mean APC and standard deviation values of hippocampus for each age group and segmentation method. We choose hippocampus because of its known vulnerability both in normal aging and in degenerative diseases such as AD. All estimated mean APC values are significantly different from zero (*t*-test, *p* < 0.01) showing that both methods are sensitive to change in all three groups. The mean differences in the APC values between the segmentation methods for each age group are all significant (paired samples t-tests, *p* < 0.01) as well indicating that SAMSEG tends to estimate smaller longitudinal changes than ASEG.

**Fig. 10.**
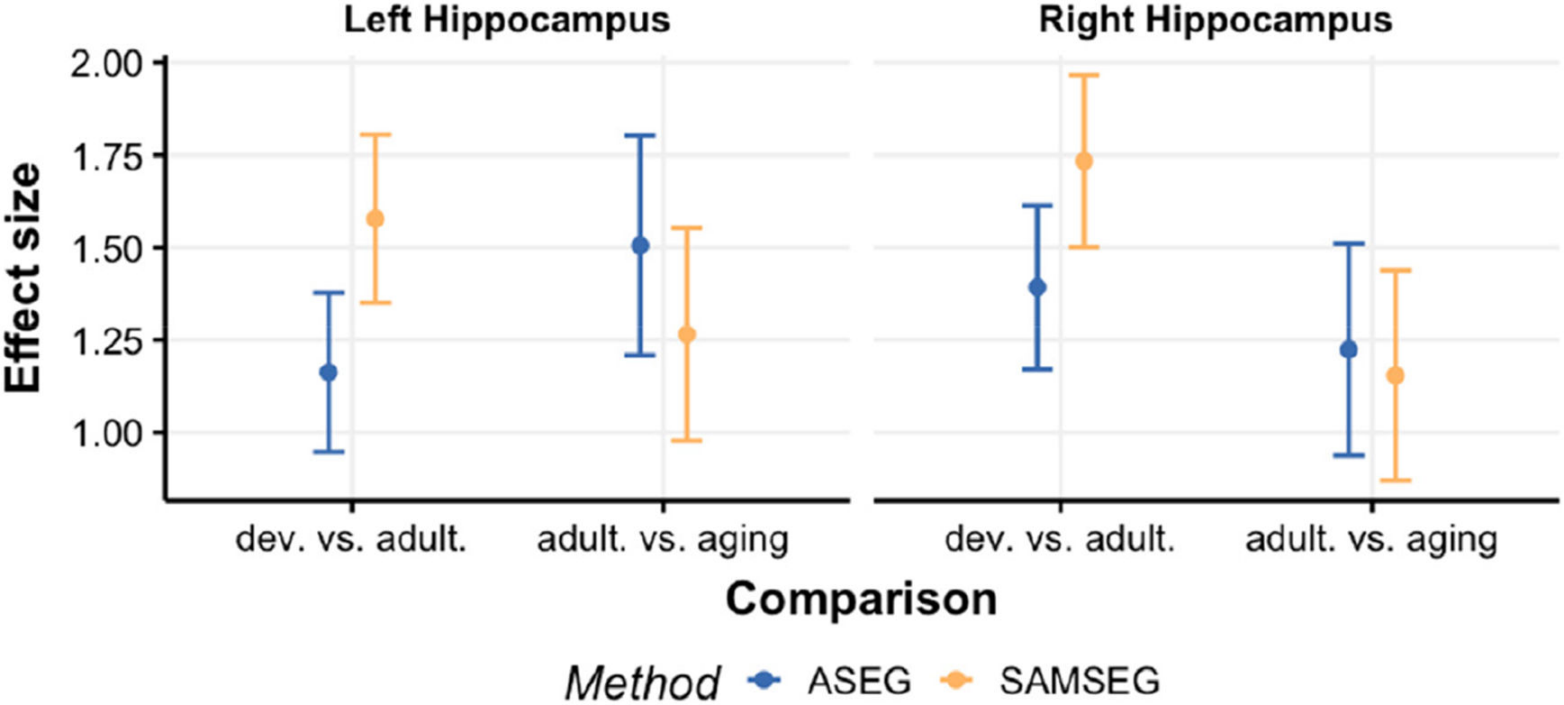
Cohen’s D effect sizes (dots) and their 95% confidence intervals (vertical bars) for development vs. adulthood, and adulthood vs. aging groups for the Avanto dataset.

Fig. 10 illustrates Cohen’s D effect sizes based on the APC values between development and adulthood, and between adulthood and aging groups for hippocampus. SAMSEG results in larger numeric effect sizes between development and adulthood whereas ASEG tends to estimate larger effect sizes for adulthood vs. aging group. However, none these differences are significant between segmentation methods.

### Clinical sensitivity

The results of the longitudinal changes indicate that SAMSEG yields lower APC values than ASEG. However, there is no ground truth whether smaller or larger changes are more accurate. We, therefore, address the clinical sensitivity using a subsample of ADNI data. For the purpose of this analysis, we only consider a hippocampus since it is the most sensitive structure for detecting AD.

**Fig. 11.**
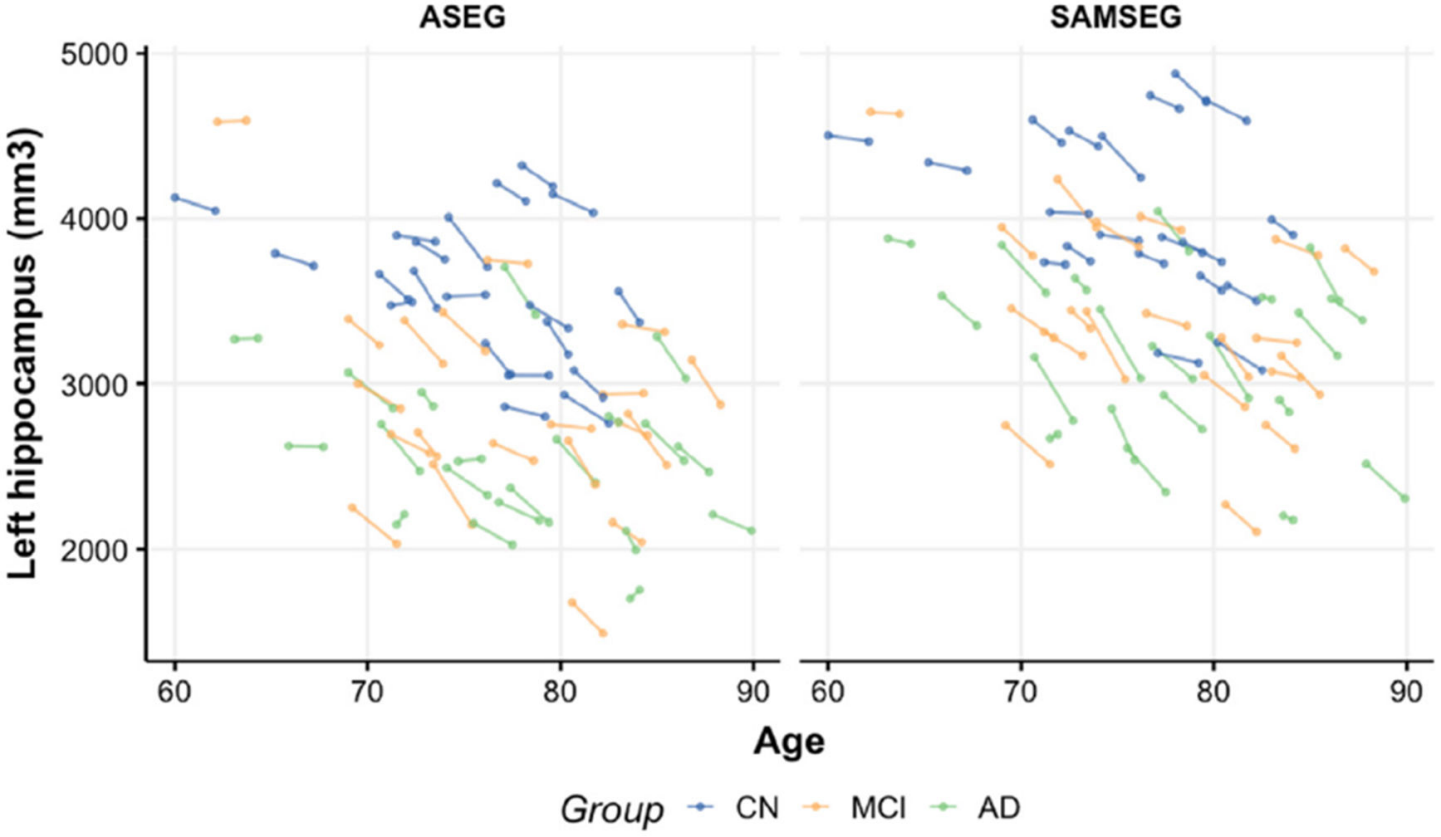
Longitudinal left hippocampus volume changes between the segmentation methods for CN, MCI and AD groups.

In Fig. 11, we present longitudinal left hippocampus volume changes for both segmentation methods. The observed differences are similar between the methods but SAMSEG yields notably larger changes in AD group. In addition, SAMSEG tends to estimate larger volumes compared to ASEG but this is consistent between the groups.

Table 3 summarizes the same group comparisons in terms of mean and standard deviation of APC values. SAMSEG estimates significantly lower APC values for CN and MCI groups but larger for AD group as compared to ASEG. Nevertheless, SAMSEG leads to the detection of significant differences in atrophy rates between all clinical groups except for the left hippocampus MCI vs. AD comparison whereas the only significant difference for ASEG is seen for the right hippocampus CN vs. MCI contrast. Generally, ASEG demonstrates larger APC variability within each group which in turn hampers the detection of significant differences between the groups when sample sizes are small.

**Fig. 12.**
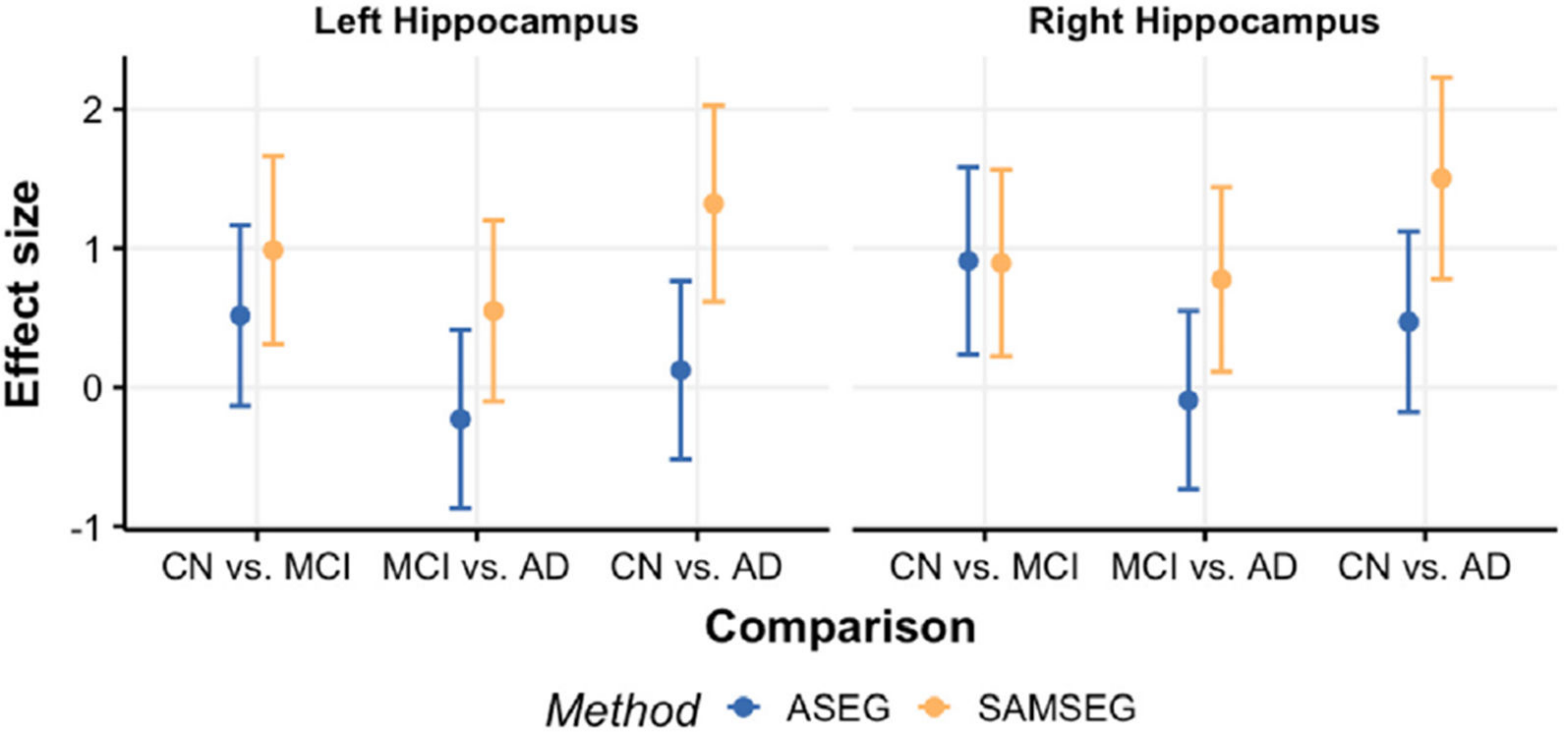
Cohen’s D effect sizes (dots) and their 95% confidence intervals (vertical bars) for the group comparisons between ASEG and SAMSEG for the left and right hippocampus.

Fig. 12 shows Cohen’s D effect sizes and their 95% confidence intervals between the group comparisons. The effects are generally larger for SAMSEG than ASEG, but none are significantly different between the segmentation methods.

**Fig. 13.**
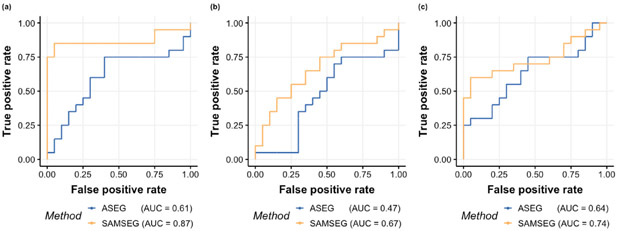
The ROC-AUC curves for classifying participants based on the APC values of the longitudinal left hippocampus estimates: (a) AD from CN, (b) AD from MCI and (c) MCI from CN.

Fig. 13 illustrates ROC-AUC curves for the classification of participants into groups based on the APC values of the left hippocampus. SAMSEG results in a larger number of correct classifications at the same or lower rate of false positives than ASEG. A very similar scenario is observed for the right hippocampus.

## 4. Discussion

The scan-rescan reliability indicates reliable volume estimation across the lifespan, scanners and segmentation methods. Slight deviations are observed for younger participants, presumably due to subtle head motion artifacts. It has previously been shown that younger age groups typically evidence increased motion artifacts, which can hinder the identification of the tissue boundaries (Blumenthal et al., 2002). Importantly, subtle motion artifacts can lead to systematic biases in automatic measurement of structural brain properties (Yendiki et al., 2014). Although different parallel imaging factors (GRAPPA) are used for the Skyra scan-rescan dataset (GRAPPA = 2 vs. GRAPPA = 1), it does not indicate sensitivity to lower signal-to-noise ratio and is comparable to the Avanto dataset. Similar effects of parallel imaging acceleration are shown by (Wonderlick et al., 2009).

**Fig. 14.**
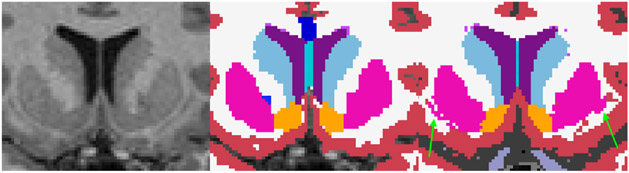
An example segmentation of the putamen structure. Left panel shows a region of MRI T1w image where putamen and claustrum are visible; center panel shows the result of ASEG segmentation; right panel shows the result of SAMSEG segmentation with green arrows pointing to the parts of claustrum structure which are segmented as putamen.

The observed average volumetric differences across the lifespan for ASEG are similar to previous reports (Jovicich et al., 2009; Morey et al., 2010). Nevertheless, SAMSEG leads to significantly higher intra-scanner volume estimation reliability for all subcortical structures and higher spatial overlap except putamen, which has significantly higher spatial overlap for ASEG. This is likely a result of SAMSEG’s probabilistic atlas, which currently does not include claustrum structure. Claustrum’s thin shape and proximity to putamen structure makes it difficult to reliably segment at common image resolutions, for example, isotropic 1 mm^3^ voxels. The probabilistic atlas used in ASEG does not include claustrum either, but it has its internal mechanism of removing it from the putamen segmentation. Fig. 14 shows an example segmentation of putamen using ASEG and SAMSEG, which outlines the inclusion of claustrum for SAMSEG.

Inter-scanner differences also support the findings of the intra-scanner reliability. Although inter-scanner differences depend on the particular comparison, SAMSEG in almost all the cases was able to estimate smaller ASPD values than ASEG. In addition, SAMSEG shows much lower variability of volumetric measures indicating improved reliability and sensitivity to detect meaningful changes. This is especially important when having small sample sizes as seen in the clinical sensitivity analysis.

Higher intra-scanner and inter-scanner reliability could come at the cost of less sensitivity to detect meaningful biological change, i.e. that SAMSEG over-regularizes. However, the present analyses of within-person longitudinal change suggest that SAMSEG does not achieve improved reliability by sacrificing sensitive to change. Longitudinal changes in hippocampal volume are detected by both methods, and the APC values are comparable. In the absence of the ground truth longitudinal changes, the present findings suggest that both methods are sensitive to changes in hippocampal volume over time.

We also mapped the lifespan trajectory of each of the structures of interest using GAMMs, taking both cross-sectional and longitudinal information into account. The segmentation differences between ASEG and SAMSEG have substantial effect on lifespan trajectories. In general, developmental trajectories are similar regardless of segmentation method, replicating previous findings (Ostby et al., 2009), although effect sizes for the hippocampus are larger for SAMSEG than ASEG when comparing development to adulthood. For adulthood and aging, however, marked differences are seen for most structures. For the hippocampus and amygdala, the ASEG results replicated earlier studies showing slight volumetric decline from young adulthood (Fjell et al., 2013), with acceleration of volume loss from the sixties, especially marked for the hippocampus. This is not observed for SAMSEG, where very little volume loss is seen before the accelerated decline in aging. For thalamus and pallidum, there are large offset effects, where the estimated volumes for the young children are much higher for ASEG, followed by a steady decline after development ends, extending throughout the rest of the lifespan. This pattern, which is in agreement with previous literature (Fjell et al., 2013), is not seen with SAMSEG. For these structures, as well as nucleus accumbens, SAMSEG yields modest decline across adulthood, with only some acceleration of volume loss in the oldest for thalamus. Interestingly, while the previously reported U-shaped trajectory for caudate (Fjell et al., 2013) is seen with ASEG, this is less evident with SAMSEG, which shows a more linear volume decline also in higher age. The implications of these findings await further explorations, but the present results show that the two segmentation methods have substantial effects on the estimated lifespan trajectories of most subcortical structures.

The longitudinal changes analyzed in the clinical setting suggest that SAMSEG tends to be more sensitive to differences in hippocampal atrophy between CN, MCI and AD groups. This is especially important for detecting the early accelerated hippocampal atrophy which is known to be one of the most sensitive biomarkers of Alzheimer’s disease (Teipel et al., 2013). Expected group differences are more consistently observed for SAMSEG than ASEG. This is likely the result of larger variability between change estimates for ASEG which in turn reduces the power to detect significant differences between the groups. Therefore, based on the current study there is evidence that ASEG might require more samples per group in order to observe the expected group differences, whereas SAMSEG already shows greater sensitivity to detect relevant changes with the relatively modest number of 20 patients in each group that we use for assessment. This is well reflected in the Cohen’s D effect sizes and ROC-AUC curves, which indicate the improved classifications based on SAMSEG’s segmentations.

We have analyzed intra-scanner reliability of participants that were not repositioned before acquiring a repeated scan. This scenario is unlikely in the clinical setting where participants are usually taken out of the scanner before acquiring another repeated scan. This, in turn, might lead to an increased measurement variability and less reliable volumetric estimates compared to what was observed in the present work. We also acknowledge that a visual rating procedure is not the most appropriate approach of pre-selecting images for the intra-scanner analysis and the study is not informative with a view to the robustness of either segmentation method in the presence of common artefacts. Finally, we performed a comprehensive evaluation of longitudinal changes and sensitivity for the hippocampus structure. The remaining subcortical structures should be addressed in addition as it is not evident that similar longitudinal trends would be present.

## Conclusions

Both whole-brain segmentation methods demonstrate high scan-rescan reliability. Although SAMSEG yields significantly lower differences between repeated measures for intra- and inter-scanner analysis, it does not compromise sensitivity to detect changes and demonstrates ability to detect clinically relevant longitudinal changes. Therefore, the method has a potential to be widely used in neuroimaging research. The present findings will also direct many researchers who have the choice between these two utilities, leading to a downstream impact in clinical studies and laying the foundation for further studies that can build on this.

## Figures and Tables

**Table 1:** A summary of MRI T1w MPRAGE acquisition parameters used for the LCBC intra- and inter-scanner datasets. For Skyra, a GRAPPA factor of 1 is used for inter-scanner analysis but 1 and 2 for intra-scanner analysis. Data of Prisma scanner is only used for inter-scanner analysis.

	Avanto	Skyra	Prisma
Field strength (T)	1.5	3	3
#slices	160	176	208
FoV (mm^2^)	240 × 240	256 × 256	240 × 256
TR (ms)	2400	2300	2400
TE (ms)	3.61	2.98	2.22
TI (ms)	1000	850	1000
FA (°)	8	8	8
Voxel size (mm^3^)	1.25 × 1.25 × 1.2	1 × 1 × 1	0.8 × 0.8 × 0.8
Bandwidth (Hz)	180	240	220
GRAPPA	1	1 (2)	2
Head coil channels	12	20	32

**Table 2:** A summary of the mean and standard deviation hippocampus APC values for the age groups, segmentation methods and datasets. Standard deviations are indicated in the parenthesis next to the mean APC values and a sample size of each group is indicated in the parenthesis next to the group name.

	Left hippocampus		Right hippocampus	
	ASEG	SAMSEG	ASEG	SAMSEG
Avanto				
development (*n* = 247)	1.18 (1.52)	0.84 (0.79)	1.23 (1.30)	0.85 (0.70)
adulthood (*n* = 159)	−0.23 (0.39)	−0.16 (0.26)	−0.22 (0.39)	−0.13 (0.23)
aging (*n* = 85)	−1.07 (0.77)	−0.76 (0.70)	−0.90 (0.78)	−0.66 (0.69)
Skyra				
development	–	–	–	–
adulthood (*n* = 119)	−0.38 (0.82)	−0.20 (0.44)	−0.38 (0.52)	−0.28 (0.33)
aging (*n* = 126)	−1.15 (1.02)	−0.77 (0.74)	−1.28 (0.92)	−0.88 (0.70)

**Table 3:** Group comparisons based on the estimated hippocampus mean APC and standard deviation values.

	Left hippocampus		Right hippocampus	
	ASEG	SAMSEG	ASEG	SAMSEG
CN	−2.18 (1.80)	−1.38 (0.76)	−1.80 (1.46)	−1.45 (0.95)
MCI	−3.26 (2.45)	−2.61 (1.59)	−3.56 (2.36)	−2.62 (1.61)
AD	−2.50 (3.98)	−3.71 (2.39)	−3.22 (4.33)	−4.21 (2.44)

## Appendix A

**Table A.1:** A summary of the mean and standard deviation (in parenthesis) ASPD values for both segmentation methods and datasets for the intra-scanner reliability analysis. Abbreviations of structure names: PT (putamen), PA (pallidum), CA (caudate), TH (thalamus), HP (hippocampus), AM (amygdala), LV (lateral ventricles) and AA (accumbens area). Names are prefixed with l- and R- to indicate left and right hemispheres respectively. Bold numbers indicate the smallest mean and standard deviation values between the segmentation methods.

	Avanto		Skyra	
	ASEG	SAMSEG	ASEG	SAMSEG
L-PT	1.15 (0.97)	**0.49 (0.40)**	1.13 (0.92)	**0.48 (0.39)**
R-PT	1.13 (0.90)	**0.49 (0.40)**	1.06 (0.85)	**0.46 (0.38)**
L-PA	1.91 (1.56)	**0.54 (0.46)**	1.89 (1.57)	**0.47 (0.40)**
R-PA	2.04 (1.73)	**0.52 (0.41)**	2.05 (1.65)	**0.49 (0.42)**
L-CA	1.05 (0.84)	**0.47 (0.40)**	1.03 (0.83)	**0.54 (0.44)**
R-CA	0.97 (0.82)	**0.49 (0.43)**	0.94 (0.81)	**0.52 (0.42)**
L-TH	1.02 (0.81)	**0.21 (0.18)**	1.16 (0.89)	**0.27 (0.22)**
R-TH	1.08 (0.88)	**0.22 (0.18)**	1.17 (0.89)	**0.28 (0.23)**
L-HP	1.33 (1.06)	**0.57 (0.52)**	1.41 (1.23)	**0.68 (0.63)**
R-HP	1.09 (0.89)	**0.54 (0.48)**	1.15 (0.93)	**0.61 (0.59)**
L-AM	3.10 (2.55)	**0.65 (0.56)**	3.40 (2.71)	**0.64 (0.54)**
R-AM	2.62 (2.12)	**0.68 (0.58)**	2.64 (2.18)	**0.65 (0.53)**
L-LV	1.00 (1.00)	**0.47 (0.42)**	0.96 (0.99)	**0.55 (0.48)**
R-LV	1.01 (1.09)	**0.51 (0.45)**	0.93 (0.92)	**0.57 (0.50)**
L-AA	4.82 (4.02)	**0.97 (0.79)**	5.66 (5.03)	**1.00 (0.89)**
R-AA	3.85 (3.25)	**1.04 (0.87)**	4.30 (3.65)	**1.09 (0.94)**

**Table A.2:** A summary of the mean and standard deviation (in parenthesis) ASPD values for both segmentation methods in the between scanner analysis. Abbreviations of structure names: PT (putamen), PA (pallidum), CA (caudate), TH (thalamus), HP (hippocampus), AM (amygdala), LV (lateral ventricles) and AA (accumbens area). Names are prefixed with l- and R- to indicate left and right hemispheres respectively. Bold numbers indicate the smallest mean and standard deviation values between the segmentation methods.

	A vs. P		A vs. S		P vs. S	
	ASEG	SAMSEG	ASEG	SAMSEG	ASEG	SAMSEG
L-PT	1.41 (1.06)	**0.83 (0.61)**	3.01 (1.33)	**1.77 (1.16)**	**1.94** (1.41)	2.35 (**1.15**)
R-PT	1.30 (0.98)	**0.87 (0.58)**	3.37 (1.95)	**1.87 (0.92)**	2.45 (1.46)	**2.10 (1.18)**
L-PA	2.96 (2.26)	**0.93 (0.67)**	2.24 (1.44)	**1.94 (0.59)**	5.83 (2.83)	**2.90 (0.87)**
R-PA	3.54 (1.93)	**1.41 (0.75)**	4.88 (2.77)	**2.66 (0.93)**	9.09 (2.87)	**3.52 (1.53)**
L-CA	3.88 (1.58)	**1.04 (0.59)**	**1.49** (1.26)	1.60 **(0.76)**	3.55 (1.75)	**1.09 (0.81)**
R-CA	2.79 (1.22)	**1.78 (0.92)**	2.10 (1.13)	**2.03 (0.73)**	1.37 (1.27)	**0.83 (0.66)**
L-TH	4.05 (1.37)	**1.75 (0.66)**	1.71 (1.26)	**1.27 (0.87)**	5.88 (1.58)	**0.79 (0.58)**
R-TH	4.03 (1.30)	**1.65 (0.78)**	2.13 (1.69)	**1.75 (1.14)**	3.28 (1.57)	**0.85 (0.52)**
L-HP	3.27 (1.38)	**3.04 (1.15)**	2.95 (1.95)	**2.42 (0.94)**	6.70 (2.66)	**4.54 (1.19)**
R-HP	5.07 (2.11)	**3.25 (1.53)**	1.77 (1.52)	**1.09 (0.84)**	6.98 (2.14)	**3.20 (1.02)**
L-AM	6.68 (3.98)	**1.80 (1.18)**	6.39 (3.70)	**1.20 (0.88)**	5.00 (2.99)	**1.47 (1.02)**
R-AM	4.79 (3.52)	**2.28 (1.29)**	4.22 (3.31)	**1.19 (0.97)**	3.63 (3.33)	**2.55 (1.43)**
L-LV	9.09 (5.00)	**2.03 (1.08)**	5.16 (2.78)	**1.84 (1.18)**	14.14 (7.19)	**3.36 (1.72)**
R-LV	8.40 (4.97)	**2.84 (1.08)**	4.46 (2.37)	**1.51 (1.23)**	12.60 (7.02)	**4.02 (1.52)**
L-AA	23.55 (10.8)	**4.69 (1.80)**	10.23 (5.82)	**2.29 (1.59)**	38.58 (15.9)	**5.42 (2.24)**
R-AA	5.15 (4.55)	**3.74 (2.22)**	4.96 (4.41)	**4.01 (2.26)**	8.34 (7.85)	**6.59 (3.54)**
